# From Clinical Presentation to the Outcome: the Natural History of PML in a Portuguese Population of HIV Infected Patients

**DOI:** 10.4021/jocmr501w

**Published:** 2011-02-12

**Authors:** Filipe Nery, Margarida Franca, Isabel Almeida, Carlos Vasconcelos

**Affiliations:** a Clinical Immunology Unit, Centro Hospitalar do Porto - Hospital Sto Antonio, Porto, Portugal

## Abstract

**Background:**

Progressive multifocal leukoencephalopathy (PML) is a demyelinating disease of the central nervous system, associated with immunosuppression states. As there are only some non-published documents concerning PML in HIV infected patients in Portugal, we pretend to characterize natural history of PML infection in a population of HIV patients.

**Methods:**

We retrospectively reviewed, from 1992 to 2009, PML cases in a population of 724 HIV infected patients followed in our institution. Clinical, biological, imagery features and outcomes were characterized.

**Results:**

Twenty-five (3.45%) patients were identified as having PML. The mean time between HIV and PML diagnosis was 20.4 months. PML was the presentation of HIV infection in 40% of the patients, and 92% had CD4 T cell count lower than 200/mm^3^. Paresis was the most common clinical presentation. No specific characteristics were found in cerebrospinal fluid and JCV DNA was positive in 3 of 7 patients. MRI revealed characteristic findings. Combined antiretroviral therapy was started or changed in 96% of the patients. Neurological condition got worse in 12 patients. From the 14 deaths, 5 were directly attributed to PML progression. Follow-up was lost in 8 patients.

**Conclusions:**

PML was the presentation of HIV infection in more than 1/3 of patients, frequently associated with advanced immunocompromise. MRI sensitivity to PML is high, and JCV DNA determination in CSF was not revealed to be sensible. PML diagnosis should be taken into account in HIV patients presenting any neurological symptoms, and HIV infection should be suspected when radiological findings suggest PML lesions even in previously healthy individuals.

**Keywords:**

Progressive multifocal leukoencephalopathy; JC virus; Human immunodeficiency virus; Demyelinating disease

## Introduction

Progressive multifocal leukoencephalopathy (PML) is a virus-induced demyelinating disease of the central nervous system, first described in 1958 by Astrom et al [1]. It has been described in different immune compromised states, typically with suppressed cellular immunity. It also occurs in immunosuppressed patients while in treatment to lymphoma and solid organ malignancies, solid organ transplant recipients, rheumatologic diseases and a variety of diseases under biologic treatment like natalizumab or rituximab [2-5]. In the 1980s, PML became an AIDS defining illness, and it is believed that nowadays 80% or more of reported PML patients are HIV positive [6, 7].

PML arises in a patient previously infected with John Cunningham virus (JCV), a polyomavirus. Primary infection occurs commonly during childhood or adolescence, and adult seroprevalence for JCV varies between 50 to 90% [8-10]. During immunosuppression, JCV can reactivate, resulting in lytic infection of oligodendrocytes in the brain and abortively infects astrocytes. Oligodendrocyte death results in demyelination and consequently a form of disease presentation that varies between patients, depending on the location of the underlying lesions [11]. Clinical presentation may be an insidious onset of cognitive impairment, weakness, visual field abnormalities, dysarthria, gait ataxia, dysphagia, peripheral neuropathy, dementia, seizures, behavioural changes and variable degrees of paresis [2, 9, 11, 12].

PML diagnosis relies on clinical presentation, neuroimaging, detection of JCV DNA in cerebrospinal fluid (CSF) and, eventually, in histological features whenever a stereotactic biopsy is done. Magnetic resonance imaging (MRI) is the gold standard neuroimaging diagnostic tool, showing typically high signal intensity areas on T2w sequence and low intensity areas on T1w sequence, without enhancement post intravenous contrast injection [13]. The presence of JCV DNA in the central nervous system (CNS), demonstrated by PCR technique, can make the final diagnosis when the clinical presentation and imagery are typical, although there is evidence that JCV can be found in CSF of HIV infected patients without PML [14, 15]. Cortical demyelinating lesions are an integral component of the pathologic process in PML, and the findings in brain biopsy of bizarre astrocytes make the final diagnosis [7, 11].

PML in HIV infected patients has a poor prognosis, and continues to be one of the deadliest opportunistic infections. Treatment of these patients remains on highly active antiretroviral therapy (HAART), based on the evidence that lowering viral HIV load, and consequently giving rise to the CD4 count, will conduct to a more effective immune defense of the host. The outcome of the patients with PML in the setting of HIV is better when HAART is started [16-18]. When associated with HAART, cidofovir might improve the outcome of the patients [19].

The aim of the study was to analyze the natural history of PML in HIV infected patients followed in our institution, from the clinical presentation features to the outcomes.

## Patients and Methods

We retrospectively reviewed an HIV population followed in our hospital between 1992 and 2009, identified as having had PML. Diagnosis of PML was based on clinical presentation, imaging, JCV PCR in CSF and biopsy findings whenever done. Data was collected as follows: HIV load, CD4 count, time between HIV and PML diagnosis, CSF biochemical and cytological characteristics, previous AIDS defining diseases, clinical presentation, imagery findings, CSF JCV PCR, brain biopsy, therapy instituted and clinical course. Statistical analysis was computed in SPSS version 17.0.

## Results

Twenty-five patients were considered eligible, as having had PML in the context of HIV infection, collected from a population of 724 HIV positive patients. Four were female (16%) and 21 were male (84%). HIV route of transmission was mainly sexual (56% of patients), followed by intravenous drug abuse (40%). In one patient, HIV transmission was attributed to bone marrow (BM) transplant in 1984. All of the 25 patients were infected with the HIV-1, and one of them had also concomitant HIV-2 infection. Eight patients (32%) were co-infected with HCV. The mean age at HIV diagnosis was 36.4 years. On average, 20.4 months after HIV diagnosis, the patients were diagnosed with PML, but with a median time of 0.75 months, due to the fact that there were cases (11 patients) with both diagnoses being made simultaneously (in 44% of the cases).

CD4 count was always available at the time of HIV diagnosis. Mean CD4 count at the time of PML presentation was of 122/mm^3^, but a notion of a consistent lower immunosuppression state is expressed by a median of 73/mm^3^. Twenty-three patients had CD4 count lower than 200/mm^3^ and two patients had CD4 count above 500/mm^3^. Only patients with PML diagnosis after 1997 (10) had HIV viral load determined, with a mean of 117951 copies/μl. Only one had less than 50 copies/ml. Six patients were under any sort of antiretroviral therapy.

Clinical presentation varied between patients, as expressed in Table 1. Motor disorders with paresis were the most common feature presentation (72%), and mainly unilateral (88.9%). Speech disorders (dysarthria, dysphasia and dyspraxia) and cognitive and behavioural disturbance followed motor disturbance, in 36% and 24% of the patients, respectively.

**Table 1:** Clinical and Biological Features and Outcome of 25 AIDS-Associated PML Patients

Patients	Sex	Route of transmission	Year of PML diagnosis	Age at PML diagnosis	Time between HIV and PML diagnosis (months)	CD4 count at onset of PML (cells/μl)	HIV viral load	Main clinical symptoms	Antiretroviral therapy before PML	Treatment started after PML	Clinical evolution	Time of follow up (months)	Main event/Outcome
1	M	Drug abuse	2009	32	3	122	13000	Convulsions, gait ataxia, unilateral paresis	None	AZT/3TC/NVP	Worse/progression	4	Dead (bacterial pneumonia)
2	M	Drug abuse	2007	45	96	76	7517	Convulsions	None	AZT/3TC/RTV/FPV	Partial recovery	31	Dead (pneumonia H1N1)
3	M	Drug abuse	1997	35	0	183	13580	Sensitivity disturbance, unilateral paresis	None	AZT/3TC/IDV	Total recovery	139	Dead (car accident with polytrauma)
4	M	Sexual	1997	43	0	73	Unk	Cognitive/behavioral disturbance, speech disorder, unilateral paresis	None	AZT/3TC/IDV	Same state	11	Lost
5	M	BM transplant	1995	18	94	94	Unk	Unilateral paresis	AZT	AZT	Worse/progression	5	Dead (PML progression)
6	M	Sexual	1999	49	0	14	> 500000	Speech disorder, unilateral paresis	None	AZT/3TC/NFV	Same state	0.75	Lost
7	M	Sexual	1992	34	3	8	Unk	Headache, sensitivity disturbance, unilateral paresis	None	None	Worse/progression	1	Dead (PML progression)
8	M	Drug abuse	1995	31	7	30	Unk	Sensitivity disturbance, unilateral paresis	AZT	AZT	Same state	2	Dead (bacterial pneumonia)
9	M	Sexual	1992	32	60	3	Unk	Sensitivity disturbance, unilateral paresis	AZT	AZT	Worse/progression	1	Dead (bacterial pneumonia)
10	F	Sexual	1993	29	47	24	Unk	Speech disorder, unilateral paresis	None	AZT	Partial recovery	1	Lost
11	M	Drug abuse	1999	43	0	127	57533	Visual and speech disorder, unilateral paresis	None	AZT/3TC/IDV	Total recovery	131	Alive
12	M	Drug abuse	1997	39	1	13	Unk	Cognitive/behavioral and visual disturbance, unilateral paresis	AZT/3TC/IDV	AZT/3TC/IDV	Same state	0.75	Dead (bacterial pneumonia)
13	M	Sexual	1998	58	0	63	Unk	Speech disorder, unilateral paresis	None	AZT/3TC/IDV	Worse/progression	1	Lost
14	M	Sexual	2008	23	0	55	76621	Convulsions	None	AZT/3TC/NVP	Partial recovery	28	Alive
15	M	Sexual	2004	27	1.5	34	Unk	Visual disorder, gait ataxia, bilateral paresis	AZT/3TC/IDV/LPV/RTV	AZT/3TC/IDV/LPV/RTV	Worse/progression	1	Lost
16	M	Drug abuse	2001	35	0	132	Unk	Convulsions	None	AZT/3TC/NFV	Same state	20	Lost
17	M	Drug abuse	2003	36	42	5	Unk	Cognitive/behavioral disturbance	None	AZT/3TC/NFV/NVP	Worse/progression	0.6	Dead (bacterial pneumonia)
18	M	Sexual	2001	48	0	14	73024	Cognitive/behavioral disturbance	None	AZT/3TC/NVP/NFV	Worse/progression	8	Dead (systemic cryptococcosis)
19	F	Sexual	2002	53	Unk	580	Unk	Cognitive/behavioral disturbance, unilateral paresis	Unknown	Continuation of the same therapy	Worse/progression	0	Dead (PML progression)
20	M	Sexual	2006	41	0	184	406979	Speech disorder, unilateral paresis	None	AZT/3TC/EFV	Worse/progression	4	Dead (PML progression)
21	M	Sexual	2001	57	5	173	Unk	Speech disorder, convulsions, unilateral paresis	None	AZT/3TC/IDV	Partial recovery	84	Dead (bacterial pneumonia)
22	M	Drug abuse	2001	30	0.5	42	Unk	Bilateral paresis	None	AZT/3TC/IDV	Partial recovery	105	Alive
23	M	Sexual	2007	44	0	111	31239	Cognitive and speech disturbance	None	AZT/3TC/FPV/RTV	Same state	1	Lost
24	F	Drug abuse	1995	40	0	175	Unk	Headache, unilateral paresis	None	AZT	Worse/progression	60	Lost
25	F	Sexual	2009	47	129	717	< 20	Speech disorder, gait ataxia	AZT/3TC/EFV	ABA/3TC/EFV	Worse/progression	11	Dead (PML progression)

M - Male, F - Female, Unk - Unknown, AZT - Zidovudine, 3TC - Lamivudine, NVP - Nevirapine, RTV - Ritonavir, FPV - Fosamprenavir, IDV - Indinavir, NFV - Nelfinavir, LPV - Lopinavir, EFV - Efavirenz

Lumbar punction was done in 19 patients. Cytology was done in all of them and biochemistry with glucose and protein analysis in 18 patients. In 6 patients leukocyte count was above the normal value, and always due to mononuclear cells. Only one patient was found to have low glucose CSF levels, and six patients, high protein levels. In all of the 19 patients that underwent CSF analysis, Gram stain was negative as was the cultural exam. JCV was searched only once in 6 patients, being positive in 2 of them. In another patient, the first four out of five determinations were negative.

All patients had brain-CT, showing hypodense lesions in the cerebral white matter, with lack of contrast enhancement in 88% of the cases.

Brain MRI was done in 16 patients, in all confirming the diagnosis, presenting characteristic findings, with T2-weighted hyperintense lesions in cerebral white matter, with hypoattenuated corresponding lesions in T1-w. The three patients with normal Brain CT scans had abnormalities in MRI, one of them with definite PML diagnosis.

Only four brain biopsies were done (patients 15, 20, 22 and 23), showing abundant macrophages, bizarre astrocytes and with nuclear inclusions in some of the cells, all samples compatible with PML diagnosis. In one of the patients (pt 15), in spite of definite PML diagnosis based on the histological finding, PCR in CSF was negative.

Therapy was continued, changed or initiated in 24 patients by the time of PML diagnosis. Before 1995 all diagnosis but one were treated with zidovudine (AZT). Age at the time of HIV and PML diagnosis, before and after introduction of cARV is expressed in Table 2.

**Table 2:** Differences Between Pre-HAART and Post-HAART Era Concerning CD4 T Cell Count, Age of Diagnosis (PML and HIV) and Death Causes

Characteristic	All (n = 25)	PML diagnosis	
		Before 1997 (n = 6)	1997 - 2009 (n = 19)
Gender	4 female, 21 male	2 female, 4 male	2 female, 17 male
CD4+ cell count at diagnosis, median (IQR), cell/μl	73 (19 - 152.5)	27 (6.75 - 114.25)	76 (34 - 173)
Age at HIV diagnosis, median (IQR), years	35.5 (30.25 - 43.0)	29 (20.75 - 34.75)	37.5 (32.75 - 45)
Age at PML diagnosis, median (IQR), years	39.0 (31.5 - 46.0)	31.5 (26.25 - 31.5)	43.0 (35.0 - 48.0)
Time between HIV and PML diagnosis, median (IQR), months	0.75 (0.0 - 33.25)	27.0 (2.25 - 68.5)	0.0 (0 - 3.5)
Death (all causes)	14	4	10
Death (attributed to PML progression)	5	2 (50%)	3 (30%)

After 1997, in spite of therapy started (or continued), in 10 patients there was a worsening of the clinical condition. Total recovery of the neurological deficits happened in only two patients (one died in a car accident, another was alive after 135 months of follow-up). Fourteen patients are known to be already dead, 5 of them due to PML progression, the others were attributed to other infections, mainly respiratory. Eight patients were lost in the follow-up.

## Discussion

In this case series we describe twenty-five HIV patients diagnosed with PML from 1992 to 2009 among 724 HIV patients, representing a PML prevalence, in the population considered, of 3.45%, concordant to that described in the literature [11, 12, 20].

HIV route of transmission of our patients is similar to that reported by the Portuguese National Coordination for HIV infection/AIDS till the end of the year 2009: 40% by intravenous drug use and 56% by sexual route [21].

PML diagnosis was the presentation of HIV infection in eleven (44%) patients. The median CD4 count in our population was 73/mm^3^, similar to that described in recent retrospective large studies [22]. In spite of that, two patients had a CD4 count above 500/mm^3^ - although a rare finding, it has already been previously described [23]. In one of those patients, PML diagnosis was probable and, in the other one, it was necessary to perform several lumbar punctions before a positive result appeared. It may be justified because techniques for determination of JCV PCR are less sensitive in patients under cARV [24].

Clinical features are quite different between patients and, although motor disorders were the most common findings, neurological presentation varied according to the cerebral region most affected by JCV infection, as expressed by imagery. As expected, CSF analysis, when performed, did not reveal any specific characteristics [25, 26]. JCV DNA determination by PCR was done in 7 patients, being positive in three of them (42.9%). There was a false negative determination in a patient with characteristic findings in MRI and histological confirmation of JCV infection. In seems that the analysis for JCV DNA early in the course of the disease may sometimes show a negative result, and that with lower immunological states, the probability of having a positive result for JCV DNA in CSF is higher [15, 26]. Three patients that were JCV positive had a CD4 count scarcely elevated than those that were JCV negative. Probably, using techniques of real-time PCR, and rising the cycling in the protocol, detection level of JCV DNA would be higher, elevating the sensitivity of the exam and diminishing the number of false negatives.

All the patients underwent a CT scan, and MRI was done in sixteen of them. Three CT scans were normal, but MRI showed characteristic findings of T2-weighted hyperintense lesions in cerebral white matter, with hypoattenuated corresponding lesions in T1-w, without mass effect, even in those patients in which CT scan was normal. MRI is the preferred imagery diagnostic tool for JCV infection, showing specific signs [13].

Antiretroviral therapy has been changing dramatically since late 1990s. This retrospective study also shows an historic perspective of antiretroviral therapy in Portugal (as it was in the most western countries). AZT was the first antiviral agent used for HIV treatment, and it is still used nowadays. It is still recommended as one of the agents to be included in therapeutic scheme due to its good penetration in CNS.

In pre-HAART era (before 1997), 3 patients diagnosed with PML were already on AZT and continued this therapy. Another two patients started on AZT. Of these five patients treated with AZT, two were lost from follow-up and the other three died. Before 1997, AZT monotherapy was definitely not an option, as was already outlined in the early 1990s [27].

We noticed that age at the moment of HIV (and subsequently PML) diagnosis is rising in the last years (cut-off being the beginning of cARV era), probably due to a change of risk behaviours for HIV transmission, as is well described elsewhere [28].

Eight patients were lost in follow-up. In the remaining seventeen patients, five died due to PML progression, nine died due to other causes and only three are still alive, one of them with total recovery and the others with partial neurological recovery.

The median time to PML-attributable death was 4.2 months, compared with 30.04 months for other-cause mortality. Patients that do not respond to treatment have a rapid course to death with a high morbidity conferred by the progression of neurological deficits. One fragility attributed to this paper, due to its retrospective character, is the loss in the follow-up of a considerable number of patients, attributed to the abandonment as well as the transfer to other institutions, concerning the residence area of the patient, what leads to an incapacity to accurately extrapolate survival rates.

In conclusion, PML among HIV infected patients, in spite of its low prevalence, must be considered whenever an HIV patient presents with neurological symptoms independently of which ones, and mainly when a lower immunosuppression state as expressed by a low CD4 count is known. When a patient presents with neurological symptoms and PML characteristic findings in MRI, an HIV test should be performed. More than one CSF sample should be performed to JCV DNA analysis if the first sample is negative. That should be done in patients under cARV, as well in those with high CD4 T cell count. PML is an AIDS defining illness that occurs more often with a CD4 count lower than 200/mm^3^. When the cause of death is PML progression, it is rapid, arising in few months, in spite of cARV started at the time of diagnosis. Finally, PML in the post-HAART era still leads to great morbimortality. It can appear even with high CD4 counts and undetectable viremia.
